# The Boy Scout

**Published:** 1910-02-26

**Authors:** 


// The Hospital

A Journal of

The Medical Sciences and Hospital Administration.

New Series. No. 158, Vol. VI. [No. 1230, Vol. XLVII.] SATURDAY, FEBRUARY 26, 1910.

### THE BOY SCOUT.

From a medical point of view the Boy Scout movement presents many interesting aspects, some of which have so far received but scant consideration. In these days when the problem of national deterioration, so eloquently and lucidly set forth by Mr. Whetham in a lately published work, is likely to receive considerable attention, it is more than gratifying to find that there are several factors which tend to counteract those favouring racial decay and which may prove comforting to the philosophic Englishman who reads the harrowing indictments framed against the community. Some of these factors are evolutionary and have always been with us. Others are legislative, and therefore fluctuant, varying as our experience of their working changes, and continually being modified in the light of such experience. Others, again, are social, and combine, in unofficial movements or measures such as the establishment of garden cities, of reformative charity associations, and of the Boy Scouts, much of the best experience and thought in dealing with problems that cannot be successfully attacked by evolutionary or legislative action. No one interested in the development of a healthy, sane population can afford to disregard these factors, or, to put it more strongly, as it deserves to be put, can allow them to become ineffective for good by neglecting actively to support them. Nor need anyone be ashamed of such support or afraid of being cajoled by sentimental objections founded on the assumption that there is no problem of national deterioration before us. We are far from believing that England is lighting her kemble-pipe at this stage of her existence, but every practitioner must be fully aware that there is a great deal of disquieting truth underlying the indictment which from time to time is presented to an astonished public and supported by a mass of overwhelming statistical evidence of physical retrogression.

One of the objections raised against the Scout movement is based on the ultra-humanitarian view that soldiering means aggressive militarism. The thoughtful may reasonably reply to this that while it is doubtless true that the movement will very materially influence the public in favour of universal military training, that can hardly be accounted an unmixed evil. No one has yet proved that the federation of the world, the placid, 'pactified union of diverse nations into one altruistic hegemony, is, from a purely scientific point of view, such a blessing as enthusiasts in the cause of peace would have us believe. The statistician, handling huge figures, may have something to say against it; and whether the theory that a heavier birth-rate of males inevitably follows the depopulation of a country emerging from a costly campaign is true or not—a matter on which no conclusive data are yet available—there seems to be evidence in favour of the view that war is a potent factor in preventing national deterioration. But it is not necessary to discuss the abstract, ethico-scientific aspects of war. It is only necessary to point out that there is an easily demonstrable fallacy in the ultra-humanitarian syllogism. No one is likely to find a word to say in favour of aggressive militarism, which inevitably favours the development of Chauvinism, bluster, and bullying; but all reasonable persons must agree that, in present circumstances, the efficient military training, the soldiering, in fact, of the male element must react favourably upon the physical and, to some extent, upon the mental improvement of the whole community. It is because we cannot blind ourselves to the advantages to be obtained by such military training that we view the Boy Scout movement with added satisfaction. Under his principals the Boy Scout learns that such training is a benefit, not necessarily a curse. He starts his work early in life, develops a liking for it, and becomes, under proper supervision, a better citizen and a better unit physically. The movement that is producing such lads all over the Empire—all over the world, in fact—is a movement that must have the cordial approval of every medical man, and for that reason medical men should take an active, participating interest in it.

There are, however, practical objections which may be brought against the system at present in vogue so far as the Boy Scout is concerned. One such is that the main mass of boyhood—the poorer class—is as yet practically untouched. It is a principle of the movement that on the field all social distinctions are dropped, and in practice there is indeed a healthy democratic spirit animating every corps. But it is worth while to point out that the distinctive Scout uniform costs money, and that this cost is a consideration to poor parents. We do not believe that the scout's uniform is essential toward the Scout's training, and we seriously question if from a hygienic point of view the present design of that uniform is desirable. The hardy Highlander, we are told, used to saturate his plaid with water and sleep in it when he wanted to be warm and cosy, but no one in his senses would think of advising the modern town-bred Scotch boy to follow the old custom. We think it highly necessary that the Central Council of the movement should insist upon a thorough medical examination of every would-be Scout before the boy is allowed to join a corps, and we would venture to suggest that it should create a medical advisory board, the members of which should be practitioners who have considerable experience in pediatrics, to help Scout masters in the task of selection and elimination. At present there are unquestionably cases in which scouting has done serious harm to children. A Scout master, with all his good sense and experience, cannot be blamed if a lad with incipient tuberculosis of the hip, or with a heart impaired by recent rheumatic fever, elects to take the field. There is naturally some rivalry among Scouts, and the movement would be worth little if there was not great enthusiasm as well. It is just in this enthusiasm and rivalry that the danger lies, and it would be largely obviated by a preliminary medical examination such as we suggest. There are many practitioners who would be glad to act as honorary consultants locally to report on such applicants, and their connection with the movement would, we think, strengthen it. We offer these suggestions with diffidence, for we believe there are already medical men on the Central Council; but the Boy Scout movement has now attained a national importance which should make members of the profession take an active interest in it, and offer, when occasion serves, their help to promote and encourage it.

